# Negative Pressure Pulmonary Edema after Reversing Rocuronium-Induced Neuromuscular Blockade by Sugammadex

**DOI:** 10.1155/2014/135032

**Published:** 2014-02-13

**Authors:** Manzo Suzuki, Toshiichiro Inagi, Takehiko Kikutani, Takuya Mishima, Hiroyasu Bito

**Affiliations:** ^1^Department of Anesthesiology, Musashikosugi Hospital, Nippon Medical School, 1-396 Kosugi-cho, Nakahara-ku, Kanagawa 211-8533, Japan; ^2^Department of Anesthesiology, Higashitotuka Memorial Hospital, 548-7 Shinano-cho, Totsuka-ku, Yokohama-shi, Kanagawa 244-0801, Japan; ^3^Department of Surgery, Higashitotuka Memorial Hospital, 548-7 Shinano-cho, Totuka-ku, Yokohama-shi, Kanagawa 244-0801, Japan

## Abstract

Negative pressure pulmonary edema (NPPE) is a rare complication that accompanies general anesthesia, especially after extubation. We experienced a case of negative pressure pulmonary edema after tracheal extubation following reversal of rocuronium-induced neuromuscular blockade by sugammadex. In this case, the contribution of residual muscular block on the upper airway muscle as well as large inspiratory forces created by the respiratory muscle which has a low response to muscle relaxants, is suspected as the cause.

## 1. Introduction

Upper airway closure after tracheal extubation is a crucial event during general anesthesia. Postoperative negative pressure pulmonary edema is an uncommon but well-described complication of upper airway obstruction [1]. Most cases of NPPE develop under the presence of laryngospasm which occurs at the time of extubation due to incomplete recovery from anesthesia, secretion, or blood irritating the vocal cord [2].

Sugammadex, a modified gamma-cyclodextrin, is a novel selective agent that can reverse rocuronium-induced neuromuscular blockade [3]. It achieves reversal of muscle relaxation by complex formation with free muscle relaxant molecules. The manufacturer recommends administration of 2 mg/kg of sugammadex after the second twitch of train of four stimulation (TOF) is obtained and extubation after the presence of TOF ratio of over 0.9 [3]. We report a case of postoperative negative pressure pulmonary edema after reversal of muscle relaxation by sugammadex due to dissociated recovery from the neuromuscular agent between the upper airway smooth muscle and respiratory muscles such as the diaphragm.

## 2. Case Report

**Figure 1:**
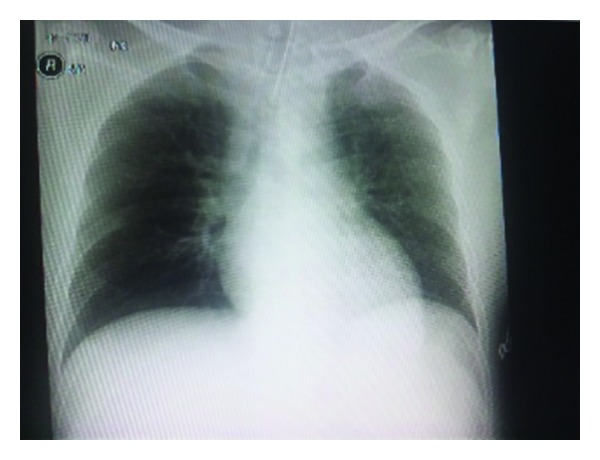
Chest roentgenogram obtained just before extubation in our patient.

A 41-year-old man with a weight of 70 kg and height of 163 cm underwent laparoscopic appendectomy for the diagnosis of acute appendicitis. He was generally healthy but had a history of asthma as a child. In the operating room, neuromuscular function was monitored using mechanomyography by train of four (TOF) built in the anesthesia monitor (S5 TM, GE Healthcare TM, Milwaukee, WI, USA). Calibration was performed at the right adductor pollicis. General anesthesia was induced by intravenous administration of propofol 120 mg and a bolus of remifentanil 0.05 mg followed by continuous infusion of remifentanil 0.2 *μ*g/kg/min; rocuronium 60 mg facilitated tracheal intubation. Bilateral transversus abdominis plane (TAP) block using 0.375% ropivacaine (20 mL, each) was performed using the ultrasound technique. General anesthesia was maintained by sevoflurane 1–1.5% and continuous infusion of remifentanil 0.1-0.2 *μ*g/kg/min, and an additional 10 mg bolus of rocuronium was given at the appearance of the second twitch of TOF. The duration of surgery was 51 minutes, and the surgery was finished uneventfully. At the time of skin closure, continuous administration of fentanyl 30 *μ*g/hr was started for postoperative pain using a patient-controlled analgesia pump (Sylinjector PCA TM, Daiken TM, Tokyo, Japan). Forty-five minutes after the final administration of rocuronium, the fourth twitch of TOF was confirmed. Sugammadex, 140 mg (2 mg/kg), was given, and infusion of remifentanil as well as propofol was discontinued. The total dose of rocuronium administered during surgery was 70 mg. The patient began spontaneous ventilation, regained consciousness, and responded to commands. The value of the T4/T1 ratio in TOF was over 90%. Chest X-ray was obtained as a routine procedure in the hospital and showed no abnormal signs (Figure 1). The tidal volume was over 400 mL and respiratory rate was over 15 breaths/min. After suctioning the sputum in the trachea and oropharynx, the trachea was extubated. Just after the extubation, the patient began to choke and developed marked respiratory depression. The airway was secured by jaw tilting, spontaneous respiration resumed with stridor, and the anesthesiologist began manual bag ventilation. Even though manual bag ventilation was possible, oxygen saturation remained at around 90%. Arterial blood gas analysis revealed marked hypercapnia and hypoxia (pH, 7.14; *P*_CO_2__, 61.8 mmHg; *P*_O_2__, 145.8 mmHg; Base Excess, −9.4, FiO_2_ = 1.0). Bilateral auscultation revealed abnormal breath sounds. During mask ventilation, frothy pink sputum was noted to be coming from the patient's mouth. A bolus of propofol 40 mg and the residual bolus of rocuronium 30 mg, which remained in the syringe, were given to reintubate.

**Figure 2:**
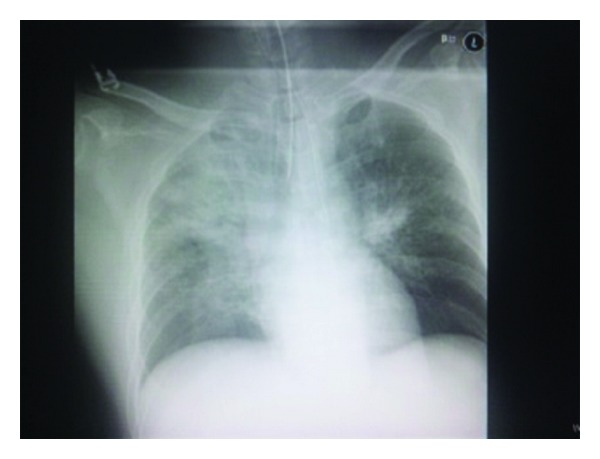
Chest roentgenogram after reintubation is shown. Marked shadow of the butterfly is seen.

The trachea was reintubated. High airway pressure was required to obtain adequate tidal volume. Chest X-ray obtained after reintubation revealed marked bilateral pulmonary edema (NPPE) (Figure 2). Arterial blood gas analysis showed remarkable hypercapnia and hypoxia (pH, 7.18; *P*_CO_2__, 60.0 mmHg; *P*_O_2__, 240 mmHg; and Base Excess, −7.0, FiO_2_ = 1.0). The patient was admitted to the ICU and received continuous positive airway pressure ventilation and administration of furosemide for two days after the surgery. The trachea was extubated two days after surgery and no clinical problems remained.

## 3. Discussion

We experienced a case of NPPE after administration of sugammadex in a healthy patient. Acute upper airway obstruction had developed after extubation. The pathophysiology of negative pressure pulmonary edema is well described as follows: a large inspiratory force in the presence of upper airway obstruction induces extremely negative intrathoracic pressure, increases blood flow into the pulmonary vasculature, and increases hydrostatic pressure and pulmonary vessel distension [4]. Among adult cases, NPPE was due to laryngospasm in more than 50% of the patients [2]. In the present case, although we suspected laryngospasm or glottic closure reflex, since we were able to secure the airway without a neuromuscular blocking agent or hypnotics, laryngospasm was more likely. Laryngospasm is defined as occlusion of the glottis secondary to contraction of laryngeal constrictors (interarytenoid, lateral cricoarytenoids, and internal and external thyroarytenoids) and is a protective reflex against mechanical or chemical internal stimuli or painful external stimuli. It involves all of the muscles of the larynx. The larynx is composed of special visceral structures that permit both voluntary and involuntary actions and is very sensitive to neuromuscular blocking agents [5]. In the present case, laryngospasm followed by NPPE developed after extubation under TOF ratio >0.9. Eikermann et al. [6] demonstrated that recovery of TOF ratio >0.9 is highly likely in the absence of neuromuscular blocking agent-induced upper airway obstruction without reversal by sugammadex. However, in the same study [6], 2 out of 70 patients presented impairment of swallowing, suggesting partial neuromuscular blockade. Herbstreit et al. demonstrated that residual neuromuscular block increases upper airway collapsibility even if the TOF ratio recovers to more than 0.8, and it does not reach the preadministration level even after TOF = 1.0 is obtained [7]. The important thing to keep in mind is that upper airway collapse is induced by the relationship between negative pharyngeal pressure by inspiratory force and upper airway patency [7]. There is a different degree of sensitivity to muscle relaxant between the upper airway muscle and diaphragm [8]. In an in vivo study in rats, after administration of sugammadex at the time of T4/T1 = 0.5, the time for recovery in respiratory function such as tidal volume was shorter than that for the time T4/T1 became 1.0 [9]. Thus, in the present patient who presented TOF > 0.9, there is still a possibility that upper airway obstruction was induced by increased upper airway collapsibility and large inspiratory forces by the diaphragm that had fully recovered from muscle relaxation by sugammadex. Thus far, the difference in recovery profile between the diaphragm and upper airway muscle by sugammadex has not been elucidated in humans. There is a possibility that rapid recovery of respiratory forces in the presence of upper airway collapsibility results in the development of NPPE.

In the present case, it is controversial whether we should have given an additional bolus of sugammadex. In critical situations such as the presence of laryngospasm, reestablishment of muscle relaxation to release the closure of vocal cord or for reintubation is required. Before reintubation, muscle relaxation was reestablished after administration of low-dose rocuronium (30 mg). Again, after anesthesia, patients who present TOF >0.9 or =1.0 after muscle relaxation do not recover from upper airway collapsibility up to the preanesthetic level [7]. An additional dose of sugammadex may have led to missing an opportunity to reestablish muscle relaxation for reintubation [10, 11].

Patients who receive sugammadex and present TOF >0.9 may develop upper airway obstruction and NPPE. We experienced a case of NPPE after reversal of rocuronium-induced muscle relaxation by sugammadex.

## References

[1] Chuang Y-C, Wang C-H, Lin Y-S (2007). Negative pressure pulmonary edema: report of three cases and review of the literature. *European Archives of Oto-Rhino-Laryngology*.

[2] Goldenberg JD, Portugal LG, Wenig BL, Weingarten RT (1997). Negative-pressure pulmonary edema in the otolaryngology patient. *Otolaryngology—Head Neck Surgery*.

[3] Sorgenfrei IF, Norrild K, Larsen PB (2006). Reversal of rocuronium-induced neuromuscular block by the selective relaxant binding agent sugammadex: a dose-finding and safety study. *Anesthesiology*.

[4] Krodel DJ, Bittner EA, Abdulnour R, Brown R, Eikermann M (2010). Case scenario: acute postoperative negative pressure pulmonary edema. *Anesthesiology*.

[5] Deepika K, Kenaan CA, Barrocas AM, Fonseca JJ, Bikazi GB (1997). Negative pressure pulmonary edema after acute upper airway obstruction. *Journal of Clinical Anesthesia*.

[6] Eikermann M, Blobner M, Groeben H (2006). Postoperative upper airway obstruction after recovery of the train of four ratio of the adductor pollicis muscle from neuromuscular blockade. *Anesthesia and Analgesia*.

[7] Herbstreit F, Peters J, Eikermann M (2009). Impaired upper airway integrity by residual neuromuscular blockade: increased airway collapsibility and blunted genioglossus muscle activity in response to negative pharyngeal pressure. *Anesthesiology*.

[8] Osawa T (2008). Different recovery of the train-of-four ratio from rocuronium-induced neuromuscular blockade in the diaphragm and the tibialis anterior muscle in rat. *Journal of Anesthesia*.

[9] Eikermann M, Zaremba S, Malhotra A, Jordan AS, Rosow C, Chamberlin NL (2008). Neostigmine but not sugammadex impairs upper airway dilator muscle activity and breathing. *British Journal of Anaesthesia*.

[10] de Boer HD, Driessen JJ, van Egmond J, Booij LH (2008). Non-steroidal neuromuscular blocking agents to re-establish paralysis after reversal of rocuronium-induced neuromuscular block with sugammadex. *Canadian Journal of Anesthesia*.

[11] Fabregat-López J, Veiga-Ruiz G, Dominguez-Serrano N, García-Martinez MR (2011). Re-establishment of neuromuscular block by rocuronium after sugammadex administration. *Canadian Journal of Anesthesia*.

